# Effects of Sleeve Gastrectomy and Subsequent Whole-Body Vibration Training on Resting Metabolic Rate and Body Composition: A Pilot Randomized Controlled Trial

**DOI:** 10.1007/s11695-026-08890-0

**Published:** 2026-08-19

**Authors:** Susana Ara-Gimeno, Alejandro Gómez-Bruton, Ana Moradell, Gabriel Lozano-Berges, María José Palacios Fanlo, Marta Sánchez-Luengo, Pilar Irún, Ángel Matute-Llorente, Ángel Lanas, Alejandro González-Agüero, José A. Casajús

**Affiliations:** 1 EXER-GENUD (Exercise-Growth, Exercise, Nutrition and Development) Research Group, University of Zaragoza, Zaragoza, Spain; 2 Faculty of Health and Sport Sciences, University of Zaragoza, Zaragoza, Spain; 3 Servicios de cirugía general, aparato digestivo y enfermedades digestivas, Hospital Clínico Universitario Lozano Blesa, Zaragoza, Spain; 4 Instituto de Investigación Sanitaria Aragón (IIS Aragón), Zaragoza, Spain; 5 Instituto de Salud Carlos III, Centro de Investigación Biomédica en Red de Enfermedades Hepáticas y Digestivas, Madrid, Spain; 6 Faculty of Medicine, University of Zaragoza, Zaragoza, Spain; 7 Faculty of Education, University of Zaragoza, Zaragoza, Spain

## Abstract

**Background:**

Sleeve gastrectomy (SG) leads to rapid weight loss, marked by reductions in fat mass (FM) and lean soft tissue (LST), contributing to a decline in resting metabolic rate (RMR). The role of early exercise and the relationship between body composition and RMR remain unclear.

**Objectives:**

To evaluate early changes in RMR and body composition after SG, explore the effects of a whole-body vibration (WBV) resistance training intervention, and examine associations between RMR and body composition.

**Methods:**

Twenty-six participants with severe obesity (61.5% female, age 40.1 ± 9.1 years, BMI 45.4 ± 4.8 kg/m²) were assessed pre-surgery (T1), 7 weeks post-surgery (T2), and 6 months post-surgery (T3). Participants were randomised to 16 weeks of WBV training or usual care. Body composition (DXA) and RMR (indirect calorimetry) were measured.

**Results:**

SG induced significant reductions in LST (-7.81 kg) and FM by T2, with a decline in absolute RMR (-357.7 ± 164.3 kcal/day). The WBV group showed mitigated loss of appendicular LST (ALST) and ALST index from T2 to T3. Over the entire study period (T1-T3), the reduction in RMR was 20% smaller in the WBV group (-430.6 vs. -536.3 kcal/day); however, this difference did not reach statistical significance (*p* = 0.085). Cross-sectionally, LST parameters were associated with RMR at all timepoints (R²=0.66–0.81), and baseline LST parameters predicted postoperative RMR.

**Conclusion:**

In this pilot trial, early WBV training attenuated losses of appendicular LST following SG. A smaller reduction in RMR was observed in the WBV group; however, this difference did not reach statistical significance and may warrant investigation in larger adequately powered trials. The predictive value of preoperative LST for postoperative RMR highlights the benefit of prehabilitation strategies to improve metabolic outcomes.

**Supplementary Information:**

The online version contains supplementary material available at https://doi.org/10.1007/s11695-026-08890-0.

## Key points

Sleeve gastrectomy induced substantial reductions in lean soft tissue (LST) and resting metabolic rate (RMR) during the first six months after surgery.

A 16-week whole-body vibration (WBV) exercise training program was safe and attenuated appendicular LST loss following surgery.

The reduction in RMR was 20% smaller in the WBV group, although between-group differences did not reach statistical significance.

Both preoperative and postoperative LST parameters were significant predictors of postoperative RMR.

**Supplementary Information:**

The online version contains supplementary material available at https://doi.org/10.1007/s11695-026-08890-0.

## Introduction

Sleeve gastrectomy (SG) is a highly effective treatment for severe obesity, driving significant weight loss through substantial reduction in both fat mass (FM) and lean soft tissue (LST) [1]. As obesity assessment increasingly extends beyond BMI alone, body composition has emerged as a clinically relevant marker of metabolic and functional health. From this perspective, an optimal body composition is characterized not only by reduced adiposity but also by the preservation of lean tissue and physical function [2, 3]. In contrast, obesity is associated with excess fat accumulation and alterations in LST, contributing to increased cardiometabolic risk, chronic disease burden, and mortality [2]. Importantly, emerging evidence indicates that low muscle mass and quality in individuals with obesity are associated with worse clinical outcomes, including frailty, disability, and increased morbidity and mortality [4, 5]. Excessive loss of Fat Free Mass (FFM) may promote the development of sarcopenia, a condition associated with functional decline and adverse health outcomes, whose risk appears to increase following metabolic and bariatric surgery (MBS) [6].

The preservation of LST, precisely quantified by dual-energy X-ray absorptiometry (DXA), is a critical concern. This compartment, comprising skeletal muscle and metabolically active organs, is a primary driver of the decline in resting metabolic rate (RMR). Its loss is therefore closely linked to the postoperative decline in RMR [7]. In addition to changes in body composition, reductions in RMR after MBS may also reflect adaptive thermogenesis, defined as a mass-independent decrease in energy expenditure in response to caloric restriction and weight loss, although its magnitude and persistence may vary across different phases of weight loss and weight maintenance [8, 9]. A lower RMR has been associated with increased susceptibility to weight gain and represents a significant obstacle to long-term weight management [9–11].

However, the determinants of RMR decline and its complex relationship with body composition are not fully understood [12]. This understanding is further complicated by methodological heterogeneity; studies employ different assessment tools, such as bioelectrical impedance analysis (BIA) or DXA, and report diverse body composition parameters (e.g., lean mass, FFM, LST), limiting direct comparisons [13]. In this context, DXA allows for a more precise and direct measurement of LST, particularly appendicular LST (ALST), a direct marker of skeletal muscle mass [14]. Focusing on these specific parameters may better elucidate the drivers of postoperative energy expenditure. Furthermore, the contribution of FM and its distribution must be considered.

In individuals with obesity, FM plays an independent role in energy expenditure [9] and a higher central body fat distribution has been associated with a higher RMR [15, 16] These observations highlight that both FM and LST contribute to RMR and that their differential loss following SG may partly explain the variability in this decline.

Exercise interventions have been proposed as strategies aiming to reduce LST loss and the associated decline in RMR [17–19], however their role in the early postoperative period after SG in individuals with severe obesity remains poorly investigated. Whole-body vibration (WBV) training is an emerging alternative for individuals with obesity and has been shown to be a safe, feasible, low-impact modality in which individuals perform static or dynamic exercises on a vibrating platform that induces rapid muscle contractions through mechanical oscillations, and has demonstrated potential in preserving skeletal muscle mass and, consequently, attenuating reductions in RMR [20, 21]. Nonetheless, its application in post-MBS populations has not yet been investigated, and thus its efficacy in maintaining these parameters remains to be established.

Therefore, the aims of the present study were (a) to evaluate early changes in RMR and body composition after SG, (b) to explore the effects of a WBV exercise intervention on these outcomes, and (c) to explore associations between RMR, LST and FM variables.

We hypothesized that SG would lead to a reduction in RMR associated with changes in body composition, and that a WBV exercise intervention would mitigate the loss of LST and potentially attenuate the decline in RMR.

## Methods

### Study Design

This study is a pilot-scale randomized controlled trial. The RCT followed the CONSORT statement [22]. The study was approved by the Clinical Research Ethics Committee of Aragón (CEICA, Spain; reference number PI22/380) and registered at ClinicalTrials.gov (NCT05695599).

Participants were randomly allocated (1:1) to two groups, the control group (CG) and the WBV group. Three assessments were conducted at predefined time points: T1) approximately 1 week before surgery; T2) seven weeks post-surgery; T3) six months post-surgery.

A detailed description of the full study protocol has been previously published [23].

#### Study Setting

The study was conducted at the EXER-GENUD Research Group, University of Zaragoza, and Hospital Clínico Universitario Lozano Blesa, Zaragoza, Spain.

### Participants

#### Sample Size

Given the pilot nature of this study, no formal a priori sample size calculation was performed for the primary outcome (RMR). The results should therefore be interpreted as exploratory.

#### Eligibility Criteria

Participants were recruited from patients scheduled to undergo SG at our institution. All patients were initially evaluated and selected for MBS by the multidisciplinary clinical team according to standard clinical criteria. These criteria reflect a population with clinically significant obesity and associated health risks requiring surgical intervention, making the inclusion of individuals with high BMI but low-fat mass (e.g., athletes) highly unlikely.

None of the participants were treated with glucagon-like peptide-1 (GLP-1) receptor agonists during the study period.

Following clinical eligibility for surgery, participants were screened for inclusion in the present study. Study-specific inclusion criteria were a BMI ≥ 40 kg/m² with or without obesity-related comorbidities, a body mass below 180 kg, and age between 18 and 50 years.

Exclusion criteria: (1) history of any type of cancer within the previous five years, (2) presence of a pacemaker or diagnosis of a cardiac condition contraindicating participation in an exercise program, (3) acute myocardial infarction within the last six months, (4) recent bone fracture, and (5) any other condition that the physician deemed a contraindication for engaging in supervised physical exercise or limitations to perform exercise or limit safe participation in, or adherence to, the supervised exercise protocol.

#### Recruitment

A total of 28 participants were recruited between February 2022 and October 2024 at the Clinico Lozano Blesa Hospital of Zaragoza. Participants meeting the inclusion criteria were informed in an individualized meeting with a nurse about the study objectives and procedures and provided written informed consent before enrolment at the hospital. After signing the informed consent form, the research team contacted each participant to initiate baseline assessments and begin the study procedures.

### Randomization Allocation Concealment and Blinding

Eligible participants were randomly assigned in a 1:1 ratio to either the WBV group or the CG, stratified by gender, using an online randomization tool https://www.sealedenvelope.com. The randomisation was conducted by one of the researchers (AGB). Group allocation was revealed to participants during their second visit. Due to the nature of the intervention, it was only the medical team who were blinded to group allocation.

### Intervention and Comparator

After SG, both groups received standard postoperative care, including recommendations to progressively increase physical activity and incorporate muscle-strengthening activities according to current clinical guidelines [24]. Patients were advised to gradually resume physical activity once surgical wounds had adequately healed, with progression guided by the surgical team. Although structured exercise is generally recommended from approximately one month after MBS [24], recent literature supports the benefits of initiating exercise earlier, within the first postoperative month [25, 26].

The WBV group participated in a supervised, progressive exercise intervention consisting of three sessions per week, each lasting 30–45 min for 16 weeks. The exercise intervention started 7 weeks post-SG, as previously published [23]. WBV was selected as a low-impact and feasible exercise modality for individuals with severe obesity during the postoperative period, with the aim to promote muscle activation while minimizing mechanical stress on the joints. In addition, WBV has been associated with osteogenic benefits [20], which may be particularly relevant in the context of the bone loss frequently observed after weight loss.

Each session included a 5–10-minute warm-up performed on a cycle-ergometer, followed by resistance training exercises performed on a WBV platform, focusing on fundamental resistance movements.

Participants were allowed to choose the time and day of sessions, provided a minimum of 24 h elapsed between sessions and no training occurred on three consecutive days.

Training was delivered on a one-to-one basis to maximize supervision, safety, and adherence during the early postoperative period. Adherence was monitored through attendance records. All sessions were conducted at the GENUD–Lab (University of Zaragoza, Spain) by a qualified researcher with a degree in Sport Sciences.

The CG received usual postoperative care only and did not participate in a structured exercise program. Their physical activity was self-managed following general recommendations to increase activity levels.

#### Session Structure

Each session was divided into three main components; a detailed description of the exercise intervention can be found elsewhere [23].

##### Warm-up (5–10 min)

Participants performed cycling on an electromagnetic cycle ergometer (Ergoline Viasprint 150p, Bitz, Alemania) at a cadence of 60–70 revolutions per minute. Resistance started at 60 W and was individually adjusted to achieve a moderate perceived exertion (score 4–5 on the OMNI scale [27]). Adjustments of watts were made in subsequent sessions if needed. Heart rate and perceived exertion were recorded.

##### WBV training (20–35 min)

Participants stood barefoot on a synchronous vertical vibration platform (Power Plate^®^ Pro5, Northbrook, IL, USA). Exercises targeted fundamental resistance movements, progressed monthly in complexity, and ensured at least one lower limb joint flexion to prevent vibration transmission to the head. Participants wore socks to prevent slipping. Exercises included calf raises, bent knee calf raises, squats, push-ups, planks and other functional movements, with progressive reduction in support from handrails over the intervention period. Training was increased by adjusting vibration time, repetitions, and reducing rest intervals. No external resistance (dumbbells or bands) was used during WBV exercises. Heart rate and perceived exertion were recorded at midpoint and end of WBV sessions.

##### Elastic band training

During the first six weeks, complementary resistance exercises using elastic bands (Thera-Band ®, Akron, EUU) were incorporated into the WBV sessions. Exercises were performed in circuit format and progressed in intensity by changing band color based on perceived exertion [28]. Elastic band exercises were incorporated during the initial phase of the intervention to provide a gradual progression of resistance exercise and facilitate adaptation to training during the early postoperative period. In addition, this phase allowed participants to develop postural control and familiarize themselves with the exercise techniques before progressing to more demanding WBV-based resistance exercises. Importantly, the elastic band component accounted for only a minor proportion of the training volume and was aimed at upper-limb strengthening. WBV remained the core component of the intervention throughout the study, with upper-body exercises subsequently performed on the vibration platform during the later stages of the programme.

### Training Monitoring and Progression

Data recorded at each session included attendance, warm-up resistance and exertion, WBV heart rate and exertion, elastic band resistance and exertion, adverse effects (e.g., pain or discomfort, dizziness), and any additional physical activity in the preceding 72 h. Monthly reviews of attendance were performed, providing feedback to participants. A minimum of two attended sessions per week was required to progress; missed sessions were compensated in subsequent weeks to ensure gradual overload.

### Measurements and Outcomes

Measurements were conducted at the GENUD-Lab at baseline, seven weeks post-SG and six-months post-SG. All assessments were performed between 08:00 and 09:00 h following an overnight fast. Participants were reminded of all pre-assessment requirements 24 h before testing, and compliance with these requirements was confirmed verbally before evaluation. Participants were also required to refrain from exercise prior to testing.

#### Body Composition and Anthropometry Assessments

Height and sitting height were measured without shoes with a stadiometer SECA 225 (SECA, Hamburg, Germany) to the nearest 0.1 cm. Waist circumference (cm) and body weight (kg) were collected with a BIA (TANITA MC780MA-N, Tanita, Tokyo, Japan). BMI was calculated by dividing body weight (kg) by height squared (m²).

Body composition variables were assessed by a Hologic Horizon A System™ QDR DXA scanner (Hologic Inc, Bedford, MA), which was calibrated before each measure. The protocol used was the standard acquisition protocol (whole body, hip and lumbar spine). In accordance with the manufacturer’s guidelines, a participant’s arm was replicated when it extended beyond the predefined scanning area.

Body composition outcomes included total body weight, FM, LST, and ALST. Regional fat distribution was also evaluated, including trunk FM. In addition, relative measures were calculated, such as the percentage of FM and LST. Specific indices were also used to better characterize body composition: the android-to-gynoid (A/G) fat ratio and the ALST index (ALST divided by height squared, kg/m²). The A/G ratio is a validated indicator of fat distribution, reflecting the proportion of fat stored in the abdominal region relative to the hips and thighs. This measure is clinically relevant as a higher A/G ratio is strongly associated with increased cardiometabolic risk, including insulin resistance, dyslipidaemia, and cardiovascular disease, and therefore serves as an important parameter for postoperative follow-up [29]. The ALST index is a recognized marker of skeletal muscle mass adjusted for body size, allowing identification of individuals at risk of sarcopenia [30]. A lower ALST index has been associated with poorer functional capacity, reduced metabolic health, and adverse outcomes after MBS [31]. Tracking changes in the ALST index is crucial for understanding the impact of surgical and rehabilitative interventions on muscle preservation and functional recovery [31].

#### Resting Metabolic Rate

RMR was assessed with by indirect calorimetry using a canopy utilizing the Cosmed Quark (Cosmed, Rome, Italy). Measurements were performed in the morning following a fast of at least eight hours, with participants resting in a supine position in a thermoneutral, quiet environment. Data were collected continuously for 30 min. To ensure accuracy and minimize the influence of initial adjustment or potential movement artifacts, only the most stable 5-minute period of measurement was selected for analysis.

Both absolute values and values normalized to body compartments were analysed. Ratios included RMR adjusted for body weight (RMR/BW), RMR relative to LST (RMR/LST), and RMR relative to FM (RMR/FM).

#### Dietary Intake and Physical Activity

Dietary intake and physical activity were assessed to provide additional information regarding lifestyle behaviours throughout the study period. Dietary intake was evaluated using a semiquantitative food frequency questionnaire (FFQ) [32] administered by a trained nutritionist, whereas habitual physical activity was assessed using the International Physical Activity Questionnaire Short Form (IPAQ-S) [33] administered by a researcher with a degree in Sport Sciences. Summary data for these variables are provided in the Supplementary Material.

### Statistical Analysis

All participants were included in the primary analysis according to the intention-to-treat (ITT) principle. A secondary per-protocol (PP) analysis was conducted including only participants who adhered to at least to 70% of the WBV intervention [23], to assess treatment efficacy under ideal conditions. Results from the ITT analysis are presented in the present manuscript, while PP findings are provided in the Supplementary Material for comparison and sensitivity assessment, in accordance with CONSORT guidelines [22].

The first part of the statistical analysis was conducted with Jamovi Version 2.6.17.0. The normality of continuous variables was assessed with the Shapiro-Wilk Test. All variables showed a normal distribution, except for the A/G fat ratio, which required logarithmic transformation to achieve normality. Descriptive data were reported as mean ± standard deviation (SD).

For the evaluation of intervention effects (SG and exercise), two separate repeated measures ANOVA models were performed: (1) a one-way repeated measures ANOVA was performed to assess the effects of SG with time as the within-subjects factor (T1 to T2) and all participants analyzed together; and (2)a mixed-design repeated measures ANOVA was conducted with Group (CG vs. WBV) as the between-subjects factor and Time (T1, T2 and T3) as the within-subjects factor to examine changes over time and differences between groups. Gender was included as a covariate in both ANOVAs to control for its potential influence on the dependent variables. In case of significant main or interaction effects, Tukey post hoc tests were applied for pairwise comparisons. Partial eta squared (η²p) was reported as an effect size index for the ANOVAs. Values of 0.01, 0.06, and 0.14 correspond to small, medium, and large effect sizes, respectively [34, 35]. ANCOVA sensitivity analyses adjusted for baseline value, age and sex were performed.

Linear regression analysis was performed to explore associations between RMR and LST, ALST and ALST index at each time point and between changes over time. A separate set of analyses examined the associations between RMR and FM variables (total FM, trunk FM, and the A/G fat ratio). Models were adjusted for gender to account for potential gender-related differences in RMR. Scatterplots with regression lines were generated for each association using R studio (2024.12.1 + 563).

## Results

### Participants

**Fig. 1:**
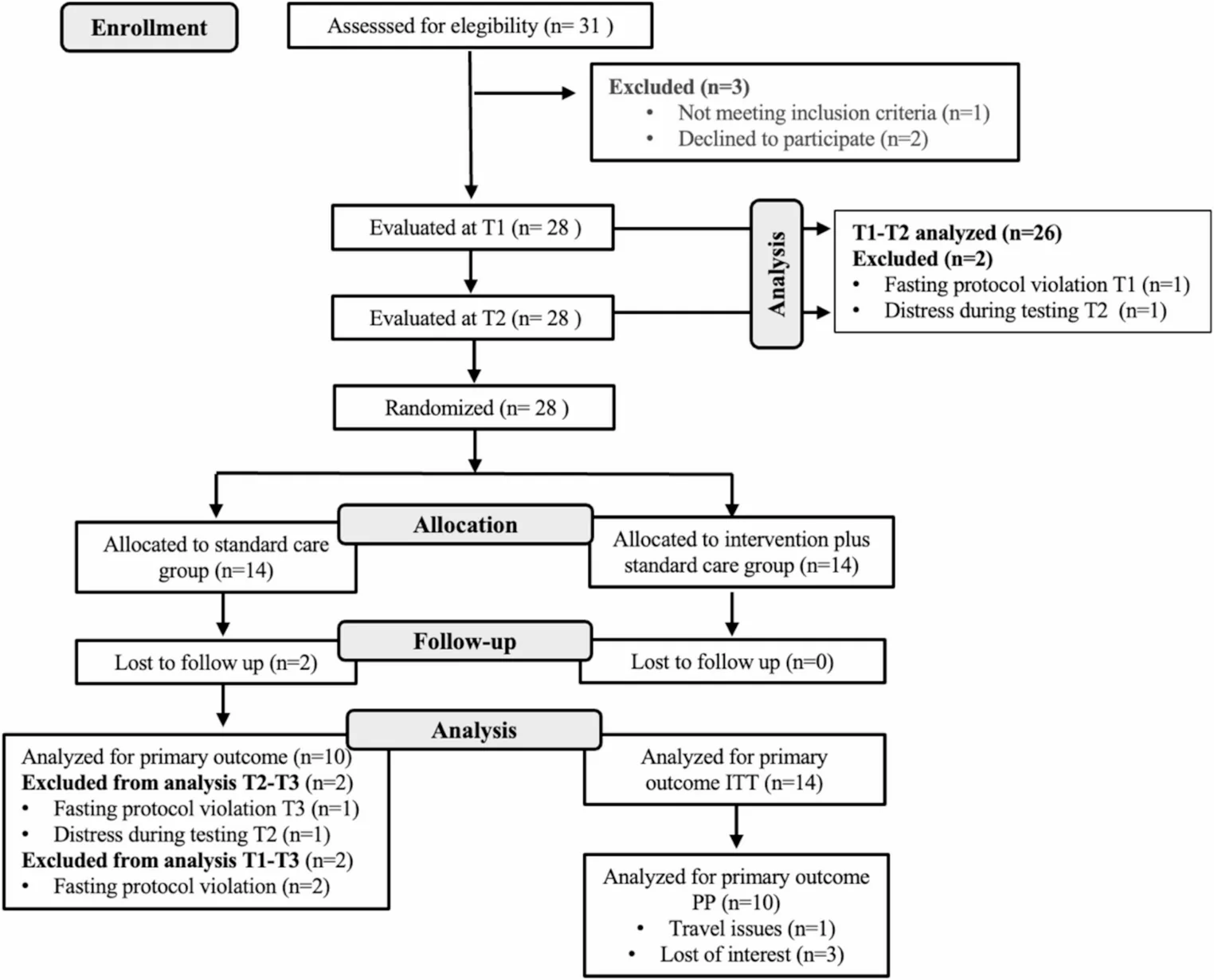
Flow of participant enrolment, allocation, follow-up, and analysis adapted from CONSORT 2025

A total of 28 patients were referred to the study over a two-year period. The first and second assessments were completed by all participants. These participants were randomized and allocated to either 16 weeks of resistance training on a WBV platform (*n* = 14) or usual care (*n* = 14). Nevertheless, 26 participants (61.5% females, mean age 40.1 ± 9.1 years; mean BMI 45.4 ± 4.8) were included in the T1-T2 analysis. Two participants were excluded from the analysis, one following early test termination due to distress at T2, and another for non-adherence to the required fasting period before the T1 evaluation. Twenty-six participants completed the assessment at T3; two dropped out, one due to pregnancy and the other because he did not attend the follow-up measurements. Although 26 participants completed the T3 assessment, two were excluded from the T2-T3 analysis: one due to an issue previously identified at T2, as the participant did not complete the test, and another for non-compliance with the pre-assessment fasting requirements at T3. For the T1-T3 analysis, both participants who violated the pre-assessment fasting protocol at T1 and T3 were excluded. The flow chart (Fig. 1) presents the number of participants in each time-point and reasons for exclusion.

The characteristics of participants and the associated comorbidities are presented in Table 1.

**Table 1:** Study participants characteristics

Parameter (Mean ± SD or *n* (%))	Baseline *n* = 26
Females (%)	16 (61.5%)
Age	40.1 ± 9.1
BMI (kg/m^2^)	45.4 ± 4.8
**Musculoskeletal disorders**	
Osteoarthritis	1 (3.8%)
Knee pain	1 (3.8%)
Low back pain	2 (7.7%)
Herniated disc	1 (3.8%)
Arthritis	1 (3.8%)
Fibromyalgia	1 (3.8%)
**Psychiatric diseases**	
Anxiety-depressive disorder	4 (15.4%)
Psychotic disorders	1 (3.8%)
Eating behaviour disorder	1 (3.8%)
Alcohol dependence	1 (3.8%)
**Respiratory diseases**	
Obstructive sleep apnoea	10 (38.5%)
Asthma	6 (23.1%)
**Metabolic disorders**	
Dyslipidaemia	2 (7.7%)
Hypertriglyceridemia	1 (3.8%)
Hypothyroidism	3 (11.5%)
**Digestive diseases**	
Dyspepsia	1 (3.8%)
Hiatal hernia	1 (3.8%)
**Cardiovascular disease**	
Arterial hypertension	5 (19.2%)

*BMI*, body mass index; *SD*, standard deviation

Mean adherence to the WBV intervention was 72.4 ± 25.3p% in the ITT analysis and 85.9 ± 9.1% in the per-protocol analysis.

### Effects of Sleeve Gastrectomy

#### Body Composition Changes

Following SG (from T1 to T2), participants showed significant reductions in body weight (−16.96 ± 3.73 kg) corresponding with a percentage of total weight loss (%TWL) of 13.1% ± 2.34, LST (−7.81 ± 2.87 kg), ALST (−3.59 ± 1.39 kg), ALST index (−1.30 ± 0.51 kg/m^2^), and FM (−8.91 ± 2.16 kg). Percentage of fat tissue declined (−0.95 ± 1.64) whereas percentage of LST increased (1.61 ± 2.42). Central adiposity decreased significantly, as shown by a marked reduction in trunk FM (−5.17 ± 1.85 kg) and waist circumference (−12.38 ± 5.99 cm). The A/G fat ratio also declined, though to a lesser extent (−0.03 ± 0.04). All results are presented in Table 2.

#### Resting Metabolic Rate Changes

RMR decreased significantly after surgery (−357.7 ± 164.3 kcal/day). RMR adjusted for body weight also declined (−0.6 ± 1.2 kcal/kg/day). No significant change was observed in RMR relative to FM (*p* = 0.790), whereas RMR relative to LST decreased significantly (−1.6 ± 2.4 kcal/kg/day).

**Table 2:** Main results before and after sleeve gastrectomy (T1-T2)

Parameter (Mean ± SD)	T1 *n* = 26	T2 *n* = 26	Δ (T1-T2)	*p*	η²ₚ
**Body composition and anthropometric variables**					
Body weight (kg)	129.42 ± 17.17	112.46 ± 15.33	−16.96 ± 3.73	< 0.001	0.96
WC (cm)	124.00 ± 11.85	111.62 ± 10.53	−12.38 ± 5.99	< 0.001	0.82
LST (kg)	67.98 ± 11.39	60.17 ± 10.41	−7.81 ± 2.87	< 0.001	0.87
LST (%)	52.50 ± 5.21	53.5 ± 5.60	1.61 ± 2.42	0.006	0.26
ALST (kg)	29.90 ± 6.11	26.3 ± 5.26	−3.59 ± 1.39	< 0.001	0.89
ALST index (kg/m²)	10.33 ± 1.39	9.03 ± 1.14	−1.30 ± 0.51	< 0.001	0.87
Fat mass (kg)	59.91 ± 10.20	50.94 ± 9.63	−8.91 ± 2.16	< 0.001	0.97
Fat mass (%)	46.42 ± 5.27	45.4 ± 6.11	−0.95 ± 1.64	0.007	0.26
Trunk fat (kg)	28.74 ± 6.11	23.58 ± 3.97	−5.17 ± 1.85	< 0.001	0.89
A/G fat ratio	0.61 ± 0.18	0.58 ± 1.17	−0.03 ± 0.04	0.007	0.26
**Resting metabolic rate variables**					
RMR (kcal/day)	2230.2 ± 346.7	1872.5 ± 282.0	−357.7 ± 164.3	< 0.001	0.83
RMR/BW (Kcal/kg/day)	17.3 ± 2.11	16.7 ± 2.00	−0.6 ± 1.21	0.023	0.19
RMR/FM (Kcal/kg/day)	38.3 ± 9.0	38.1 ± 10.0	−0.2 ± 3.5	0.790	< 0.01
RMR/LST (Kcal/kg/day)	33.0 ± 2.7	31.4 ± 2.7	−1.6 ± 2.4	0.002	0.31

*A/G*, Android/Gynoid ratio; *ALST*, Appendicular lean soft tissue;* BW,* Body weight; cm, centimetres; *FM,* Fat mass; *kcal*, kilocalories; *kg*, kilograms; *LST*, Lean Soft tissue; *η²ₚ*, Partial eta square; *p*, significance level; *RMR*, Resting Metabolic Rate; *SD*, standard deviation; *SG*, Sleeve Gastrectomy; *T1*, one week before surgery; *T2*, seven weeks post-surgery; *T3*=, six months post-surgery; *WC*, Waist circumference; Δ, change

**Fig. 2:**
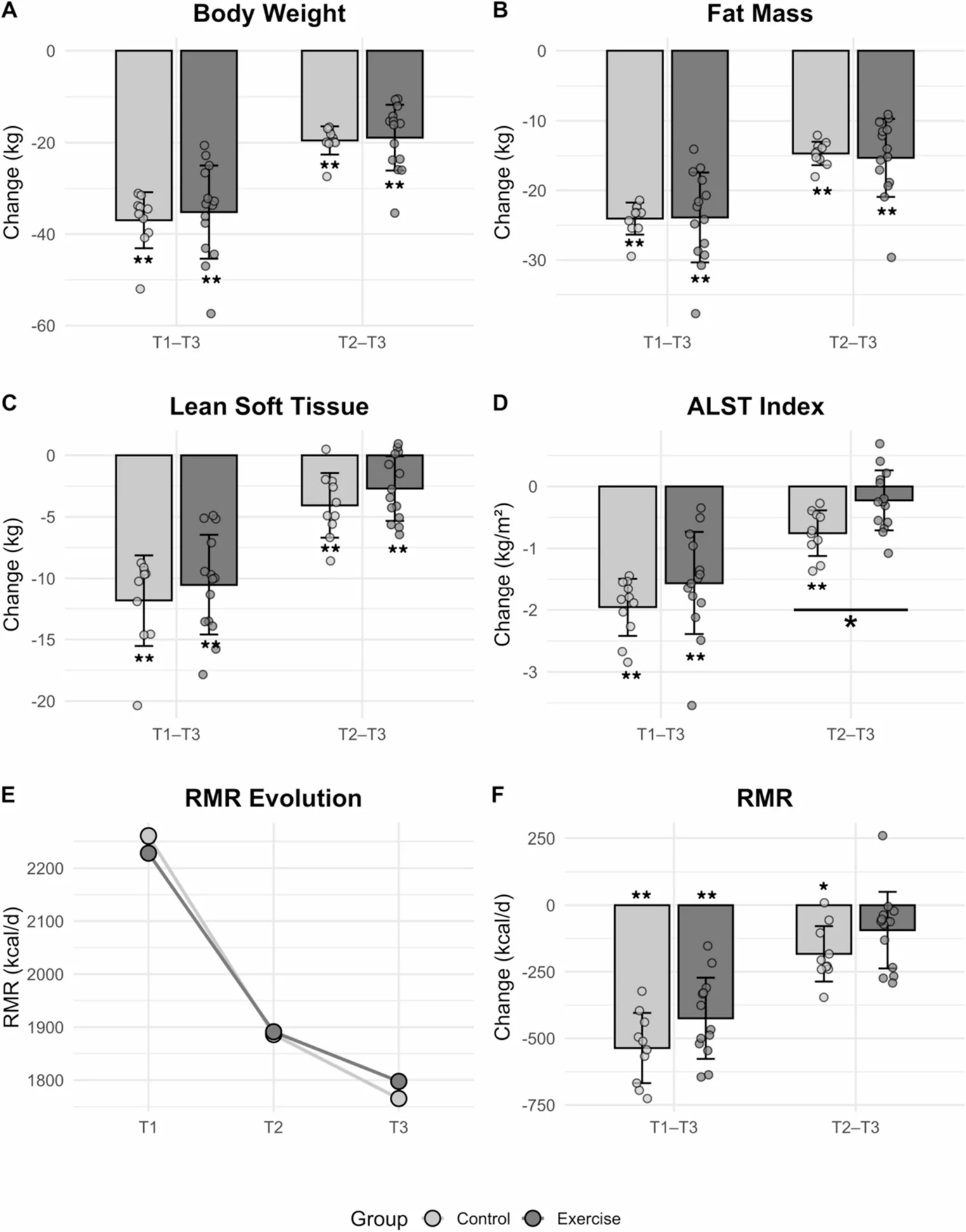
Changes in body composition and RMR after sleeve gastrectomy and WBV training. **A**. Body weight changes; **B**. Fat mass changes; **C**. Appendicular lean soft tissue changes. **D**. Appendicular lean soft tissue index changes. **E**. Resting metabolic rate evolution; **F**. Resting metabolic changes. T1, one week before surgery; T2, seven weeks post-surgery; T3, six months post-surgery; Δ, change; *Within group changes (p < 0.050); **Within group changes (p < 0.001)

### Effects of the Exercise Intervention (T2-T3)

In the ITT analysis, percentage of total weight loss (%TWL) was similar between groups (*p* = 0.292), with the CG achieving − 17.7% ± 2.75 and the WBV group − 16.1% ± 4.22. Both groups experienced significant reductions (*p* < 0.001) in most body composition variables during the intervention period (T2-T3). Detailed results are presented in Table 3. Figure 2 illustrates the primary outcomes.

**Table 3:** Effects of WBV exercise intervention (T2-T3). ITT analysis

Parameter (Mean ± SD)	WBV Group *n* = 14				Control Group *n* = 10				GxT	η²_*p*_
	T2	T3	Δ (T2-T3)	η²_*p*_	T2	T3	Δ (T2-T3)	η²_*p*_		
**Body composition and anthropometric variables**										
Body weight (kg)	115.26 ± 18.63	96.46 ± 13.77	−18.90 ± 7.20**	0.88	110.64 ± 10.39	91.13 ± 10.06	−19.51 ± 3.09**	0.98	0.804	< 0.01
Waist Circumference (cm)	111.00 ± 10.70	95.71 ± 8.22	−15.29 ± 7.57**	0.82	112.30 ± 9.49	97.00 ± 6.09	−15.30 ± 6.65**	0.86	0.996	< 0.001
Lean soft tissue (kg)	61.50 ± 10.66	58.80 ± 10.15	−2.70 ± 2.62**	0.53	59.03 ± 9.85	54.96 ± 10.15	−4.07 ± 2.63**	0.73	0.222	0.07
Lean soft tissue (%)	53.50 ± 6.28	61.11 ± 7.80	7.60 ± 3.34**	0.85	53.51 ± 6.28	59.94 ± 5.59	6.90 ± 2.03**	0.93	0.563	0.02
Appendicular LST (kg)	26.90 ± 5.17	26.20 ± 5.10	−0.63 ± 1.38	0.19	25.50 ± 5.59	23.40 ± 5.51	−2.10 ± 1.11**	0.83	0.006	0.28
ALST index (kg/m²)	9.17 ± 1.29	8.95 ± 1.16	−0.22 ± 0.48	0.18	9.10 ± 1.07	8.35 ± 1.17	−0.75 ± 0.37**	0.82	0.008	0.28
Fat mass (kg)	53.33 ± 12.82	37.01 ± 10.29	−15.32 ± 5.61**	0.89	50.00 ± 3.03	35.31 ± 4.16	−14.69 ± 1.69**	0.99	0.734	0.01
Fat mass (%)	45.19 ± 6.90	38.18 ± 8.09	−7.01 ± 2.72**	0.88	52.33 ± 12.82	39.11 ± 5.82	−6.47 ± 1.43**	0.96	0.569	0.01
Trunk fat (kg)	23.31 ± 4.95	15.57 ± 4.27	−7.73 ± 2.81**	0.89	24.11 ± 2.45	16.35 ± 2.76	−7.77 ± 1.74**	0.96	0.976	< 0.001
A/G fat ratio	0.55 ± 0.12	0.48 ± 0.12	−0.07 ± 0.04**	0.77	0.61 ± 0.13	0.54 ± 0.09	−0.08 ± 0.06**	0.61	0.687	0.01
**Resting metabolic rate variables**										
RMR (kcal/day)	1891.2 ± 281.6	1797.6 ± 257.1	−93.6 ± 143.8	0.31	1931.0 ± 332.8	1749.1 ± 266.7	−182.7 ± 104.0*	0.77	0.109	0.11
RMR/BW (Kcal/kg/day)	16.6 ± 2.4	18.8 ± 2.5	2.2 ± 1.6**	0.68	17.4 ± 1.6	19.2 ± 1.7	1.8 ± 1.2*	0.71	0.487	0.02
RMR/FM (Kcal/kg/day)	38.3 ± 12.6	53.4 ± 23.1	15.1 ± 11.7**	0.85	38.9 ± 8.1	50.7 ± 13.2	11.8 ± 6.4**	0.91	0.310	0.05
RMR/LST (Kcal/kg/day)	31.0 ± 2.9	30.8 ± 2.3	−0.2 ± 2.6	0.01	32.8 ± 1.6	32.1 ± 2.3	−0.7 ± 2.3	0.10	0.631	0.01

*A/G*, Android/Gynoid ratio; *ALST*, Appendicular lean soft tissue; *BW*, Body weight; cm, centimetres; *FM*, Fat mass; *GxT*, Group by time interaction; *η²ₚ*, Partial eta square; kcal, kilocalories; kg, kilograms; *LST*= Lean Soft tissue; *p*, significance level; *RMR*, Resting Metabolic Rate; *SG*, Sleeve Gastrectomy; *T2*, seven weeks post-surgery; *T3*, six months post-surgery Δ, change; *Within group changes (*p* < 0.05); **Within group changes (*p* < 0.001)

**Fig. 3:**
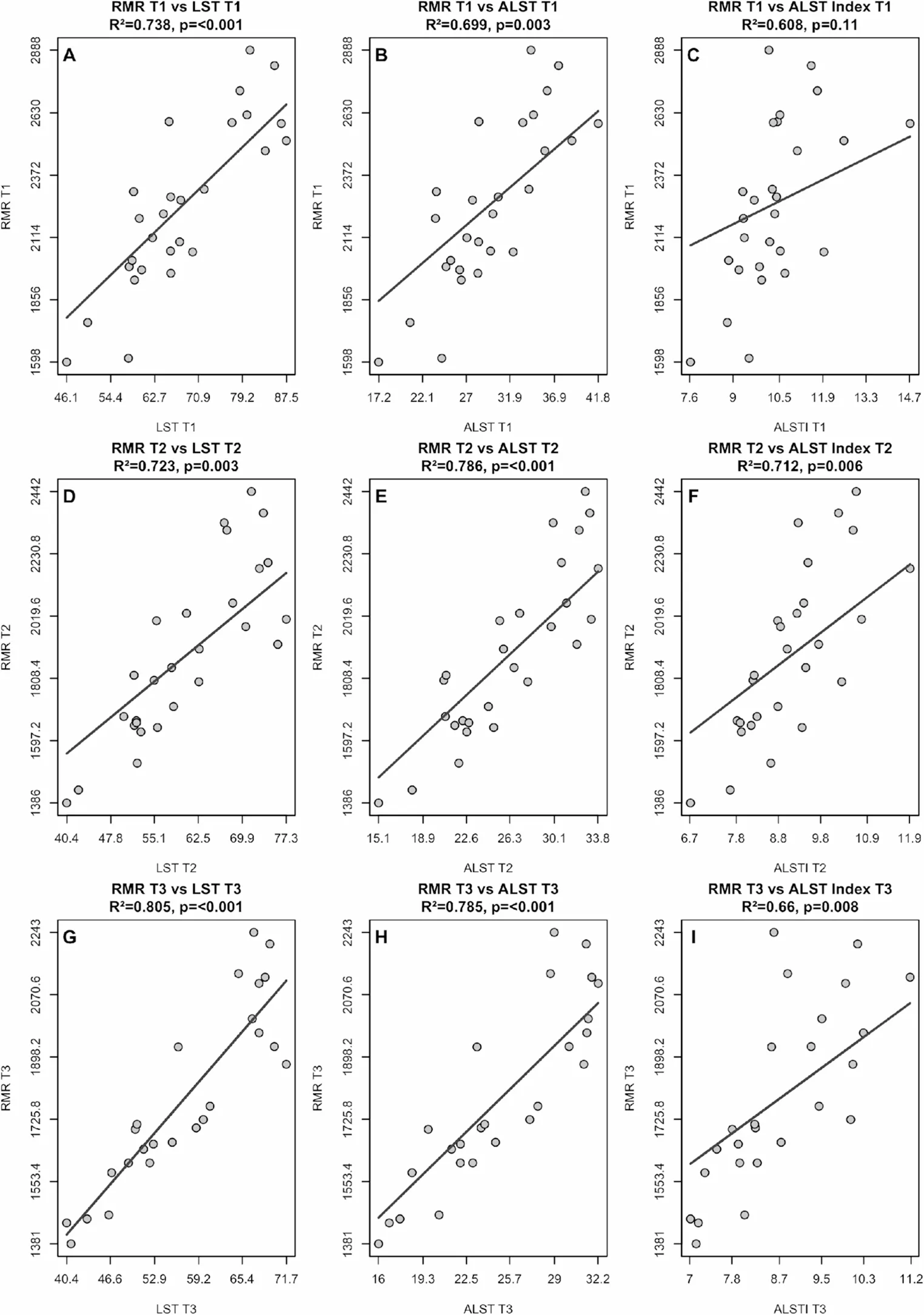
Relationship between RMR and total LST variables across study time points, adjusted for gender. (**A**) RMR T1 vs. LST T1; (**B**) RMR T1 vs. ALST T1; (**C**) RMR T1 vs. ALST index T1; (**D**) RMR T2 vs. LST T2; (**E**); RMR T2 vs. ALST T2; (**F**) RMR T2 vs ALST index T2 (**G**) RMR T3 vs. LST T3; (**H**) RMR T3 vs. ALST T3; (**I**) RMR T3 vs. ALST Index T3. ALST, Appendicular lean soft tissue; ALST Index, Appendicular Lean Soft Tissue Index; LST, Lean Soft Tissue; p, p-value; RMR, Resting Metabolic Rate; R², coefficient of determination T1, one week before surgery; T2, seven weeks post-surgery; T3, six months post-surgery

A significant group-by-time interaction was observed for the ALST (*p* = 0.006) and ALST index (*p* = 0.008) with the CG group showing a greater decrease in both parameters. Adjusted analyses showed significant group differences at T3 for ALST (F = 7.868, *p* = 0.011, η²*p* = 0.293) and ALST index (F = 6.855, *p* = 0.008, η²*p* = 0.318), whereas no significant adjusted effects were observed for the remaining outcomes (Supplementary Material Table S4).

Regarding RMR, no significant change was observed in the WBV group (−93.57 ± 143.84, *p* = 0.057) whereas the CG experienced a significant decrease (*p* = 0.001). However, no significant group-by-time-interactions were found.

Both groups exhibited significant and comparable increases in RMR adjusted for body weight (WBV: 2.47 ± 1.52; CG: 2.03 ± 0.98) and for FM (WBV: 17.90 ± 9.25; CG: 18.20 ± 8.54). In contrast, RMR relative to LST remained unchanged in both groups. No significant group-by-time interactions were observed for these variables. In the PP analysis, the results were generally consistent with the ITT findings. (Supplementary Material Table S.2)

### Effects of the SG and Exercise Intervention (T1-T3)

At six months after MBS (T3), %TWL was comparable between groups (*p* = 0.154), with the CG achieving − 29.0% ± 3.53 and the WBV group − 26.4% ± 4.54. Both groups showed significant reductions in all body composition variables (*p* < 0.001), with no significant group by time interactions. RMR decreased significantly in both groups (WBV: −430.6 ± 148.2 kcal/day; CG: −536.3 ± 131.9 kcal/day, *p* < 0.001), no significant group by time interaction was observed, *p* = 0.085. Sensitivity ANCOVA analyses adjusted for baseline value, age, and sex showed a significant group effect for RMR at T3 (F = 4.866, *p* = 0.040, η²*p* = 0.204). Although the adjusted group effect for ALST did not reach statistical significance, a moderate effect size was observed (F = 4.107, *p* = 0.057, η²*p* = 0.178). No significant adjusted effects were found for the remaining body composition outcomes. Detailed results are presented in Table 4 and Supplementary Table S5.

**Table 4:** Changes from baseline (T1-T3). ITT analysis

Parameter (Mean ± SD)	WBV Group *n* = 14		Control Group *n* = 10		GxT	η²*p*
	Δ (T1-T3)	η²*p*	Δ (T1-T3)	η²*p*		
**Body composition and anthropometric variables**						
Body weight (kg)	−35.19 ± 10.19**	0.93	−36.97 ± 6.14**	0.96	0.629	0.01
Waist Circumference (cm)	−26.79 ± 7.58**	0.93	−29.00 ± 7.44**	0.85	0.484	0.02
Lean soft tissue (kg)	−10.53 ± 4.07**	0.79	−11.83 ± 3.70**	0.92	0.432	0.04
Lean soft tissue (%)	8.29 ± 3.05**	0.89	7.98 ± 1.79**	0.73	0.773	< 0.01
Appendicular LST (kg)	−4.55 ± 2.43**	0.79	−5.41 ± 1.54**	0.94	0.296	0.05
ALST index (kg/m²)	−1.56 ± 0.83**	0.79	−1.97 ± 0.49**	0.95	0.180	0.08
Fat mass (kg)	−23.89 ± 6.46**	0.94	−24.05 ± 2.30**	0.99	0.940	< 0.001
Fat mass (%)	−8.00 ± 2.62**	0.91	−7.44 ± 1.82**	0.94	0.912	< 0.01
Trunk fat (kg)	−12.39 ± 3.39**	0.94	−13.84 ± 2.77**	0.97	0.278	0.05
A/G fat ratio	−0.11 ± 0.07**	0.72	−0.10 ± 0.08*	0.66	0.431	0.03
**Resting metabolic rate variables**						
RMR (kcal/day)	−430.6 ± 148.2**	0.90	−536.3 ± 131.9**	0.95	0.085	0.13
RMR/BW	1.7 ± 1.3	0.66	1.4 ± 1.2	0.60	0.551	0.02
RMR/FM	15.15 ± 13.78**	0.83	11.70 ± 7.53**	0.89	0.334	0.04
RMR/LST	−1.50 ± 2.17	0.34	−2.30 ± 2.29	0.53	0.392	0.03

*A/G*, Android/Gynoid; *ALST*, Appendicular lean soft tissue; *BW*, Body weight; cm, centimetres; *FM*, Fat mass; *GxT*, Group by time interaction; *η²ₚ*, Partial eta square; kcal, kilocalories; kg, kilograms; *LST*, Lean Soft tissue; p, significance level, *RMR*, Resting Metabolic Rate; SG, Sleeve Gastrectomy; *T1*, 1 week before surgery; T3, 6 months post-surgery Δ, change; %, Percentage; *Within group changes (*p* < 0.050); **Within group changes (*p* < 0.001)

### Associations Between Resting Metabolic Rate and Lean Soft Tissue Variables. ITT Analysis

#### RMR and Lean Soft Tissue Parameters Across Time Points

Figure 3 presents the associations at different timepoints between LST and RMR. At baseline (T1), strong positive associations were observed between RMR and LST (β = 21.36, R² = 0.738, *p* < 0.001), as well as with ALST (β = 31.91, R² = 0.699, *p* = 0.003). The association between RMR and ALST index showed a positive but non-significant trend (β = 63.54, R² = 0.608, *p* = 0.112).

At T2, RMR remained significantly correlated with LST (β = 16.58, R² = 0.723, *p* = 0.003), ALST (β = 37.29, R² = 0.786, *p* < 0.001) and with ALST index (β = 109.70, R² = 0.712, *p* = 0.006).

At T3, these relationships persisted, with RMR being associated with LST (β = 22.45, R² = 0.805, *p* < 0.001) and ALST (β = 36.69, R² = 0.785, *p* < 0.001).

#### Changes in RMR and Lean Soft Tissue Parameters

**Fig. 4:**
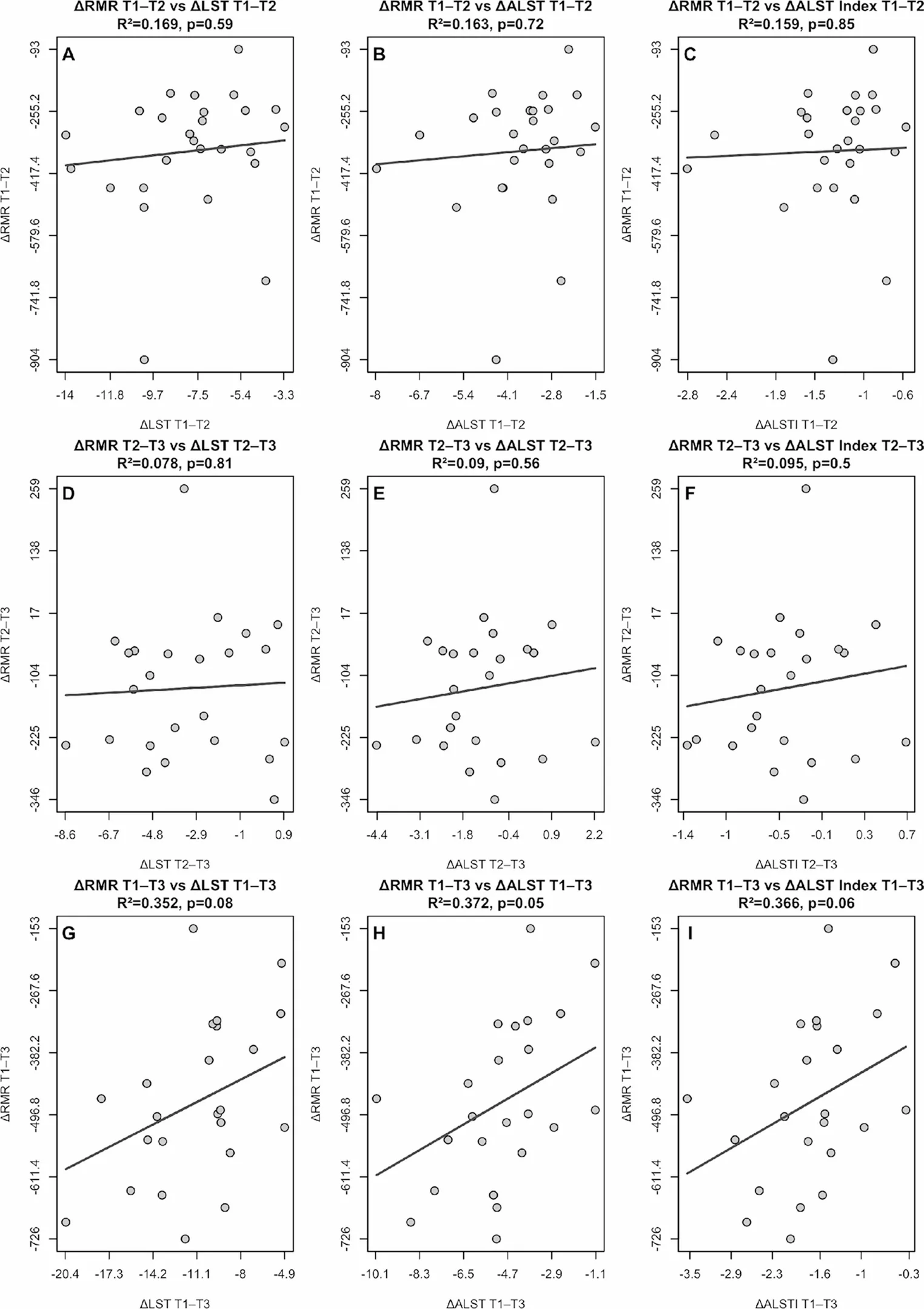
Relationship between changes in RMR and changes in LST parameters across study time points, adjusted for gender. (**A**) ΔRMR T1–T2 vs. ΔLST T1–T2; (**B**) ΔRMR T1–T2 vs. ΔALST T1–T2; (**C**) ΔRMR T1–T2 vs. ΔALST index T1–T2; (**D**) ΔRMR T2–T3 vs. ΔLST T2–T3; (**E**) ΔRMR T2–T3 vs. ΔALST T2–T3; (**F**) ΔRMR T2–T3 vs. ΔALST index T2–T3. (**G**) ΔRMR T1–T3 vs. ΔLST T1–T3 (**H**) ΔRMR T1–T3 vs. ΔALST T1–T3 (**I**) ΔRMR T1–T3 vs. ΔALST index T1–T3; ALST= Appendicular lean soft tissue; ALST Index =Appendicular Lean Soft Tissue Index; LST=Lean Soft Tissue; p= p-value; RMR= Resting Metabolic Rate; R²= coefficient of determination. T1= 1 week before surgery; T2= 7 weeks post-surgery; T3= 6 months post-surgery

When analysing changes over time, no significant associations were found between variations in RMR and corresponding changes in LST, ALST, or ALST index from T1–T2 or T2–T3 (Fig. 4**)**.

However, from baseline to follow-up (T1–T3**)**, there was a tendency for positive associations between the change in RMR and changes in lean tissue parameters, including LST (β = 13.37, R² = 0.352, *p* = 0.079), ALST (β = 26.36, R² = 0.372, *p* = 0.054), and ALST index (β = 73.25, R² =0.366, p = 0.060) (Fig. 4).

####  Baseline Lean Soft Tissue as a Predictor of RMR

Figure S1 shows LST measured at T1 as a predictor of RMR at T2 (β = 15.82, R² = 0.726, *p* = 0.002) and T3 (β = 17.77, R² = 0.765, *p* < 0.001). Similarly, ALST and ALST index at T1 predicted RMR at T2 (ALST: β = 29.36, R² = 0.766, *p* < 0.001; ALST index: β = 69.33, R² = 0.660, *p* = 0.027) and T3 (ALST: β = 29.03, R² = 0.756, *p* < 0.001; ALST index β = 64.24, R² = 0.608, *p* = 0.039).

### Association Between Resting Metabolic Rate and Fat Variables. ITT Analysis

Cross-sectional analyses showed that trunk FM was significantly associated with RMR at baseline (β = 19.789, R² = 0.633, *p* = 0.044). However, total FM and A/G fat ratio showed no significant associations at any timepoint in cross-sectional analyses.

Longitudinal analyses demonstrated that changes in fat trunk mass were significantly associated with RMR changes during both short-term (T1-T2: β = 38.456, R² = 0.314, *p* = 0.032) and long-term periods (T1-T3: β = 21.271, R² = 0.422, *p* = 0.021).

No significant association was observed between RMR and changes in total FM or A/G fat ratio (Figures S2 and S3).

### Adverse Effects

No serious adverse events or intervention-related complications were reported during the study period. Some participants experienced transient dizziness or fatigue during the initial sessions, which were managed by extending recovery periods when necessary. These symptoms resolved spontaneously, did not require medical attention, and did not lead to session interruption or withdrawal from the intervention.

## Discussion

This study aimed to examine changes in RMR and body composition following SG, as well as the effects of an exercise intervention based on WBV and to elucidate the association between RMR and LST and FM parameters. The key findings demonstrate that SG induces significant, rapid losses in both FM and LST accompanied by a substantial decline in RMR indicative of metabolic adaptation. While the WBV intervention showed limited additional benefits in body composition variables, it provided a potential benefit in attenuating the ALST index and showed a non-significant trend towards preserving absolute RMR in the longer term. Crucially, cross-sectional analyses confirmed LST as a predictor of RMR at all time points, both before and after surgery.

### Metabolic and Body Composition Shifts After Sleeve Gastrectomy

Consistent with the established literature [1, 36–38] our participants experienced a marked reduction in body weight, FM and central adiposity. This was evidenced by the marked decreases in waist circumference, trunk FM, and to a lesser extent, the A/G fat ratio. A critical finding was the significant loss of LST (−7.81 kg) within the first seven weeks after surgery, which constituted 46.1% of the total weight lost. This rate of LST loss is notably rapid, representing approximately 70% of the total loss observed at six months post-surgery. This finding aligns with a systematic review and meta-analysis [1], which identified the first three months after surgery as a vulnerable phase where 50–66% of the annual FFM loss occurs. Furthermore, another study [36] exploring acute and long-term changes after SG reported an intense loss of 5.7 kg (9.7%) in the first month, underscoring the particularly severe catabolism in the immediate postoperative period.

Concurrently, we observed a significant decrease in absolute RMR (−357.7 ± 164.3 kcal/day), a consistent result with the literature [10, 12, 19, 39, 40]. A decrease of 300–400 kcal/day six months after surgery has been reported [10, 39, 40]. Our data also reveal that this metabolic slowdown is not solely a function of reduced mass. The persistence of a significantly lower RMR after adjusting for both body weight (−0.57 ± 1.21 kcal/kg/day) and LST (−1.61 ± 2.42 kcal/kg LST/day) indicates a profound reduction in the metabolic efficiency of the remaining tissue. This means that after surgery, each kilogram of metabolically active LST burns fewer calories than would be predicted, this adaptive thermogenesis is specifically tied to the LST compartment, a conclusion strongly supported by the finding that RMR adjusted for FM remained unchanged from T1-T2 (*p* = 0.790). The stability of the RMR/FM ratio could be explained by the distinct physiological roles of these tissues. Consequently, the observed adaptive thermogenesis is not a uniform process across all tissues but is a specific downregulation of the metabolic activity of the LST. This is corroborated by a meta-analysis of gastric bypass patients, which demonstrated a significant reduction in RMR relative to FFM of 1.95 kcal/kg/day at 12 months post-surgery [41].

### Role of Exercise Combined with Sleeve Gastrectomy

Seven weeks after SG (T1-T2), while both groups continued to lose weight and FM similarly from T2 to T3, a significant group-by-time interaction was found for the ALST and ALST index. The CG experienced a greater decrease, suggesting that WBV may have partially mitigated the loss of ALST, which is a key component of functional strength. Results are particularly noteworthy within the context of the existing literature. A recent overview of systematic reviews [42] has concluded that interventions shorter than six months often fail to produce additional significant effects on body weight, with inconclusive evidence regarding the preservation of FFM, with no studies able to completely prevent its loss. Bellicha et al. [43] conducted a meta-analysis, which showed that exercise during the first post-operative year prevents an average of only 1 kg of FFM loss, a small fraction of the total lost. Consistent with our findings, when compared to the CG, the WBV group lost 1.37 kg less LST than the CG from T2-T3 and 1.30 kg from T1-T3. The evidence on the ALST index specifically remains limited. A previous trial [44] with 16 weeks of exercise starting one month post-surgery reported no intergroup differences. In contrast, Boppre et al. [45], who conducted an 11-month intervention, showed that exercise was effective in mitigating the loss of lower-limb and ALST. The positive results regarding ALST in the present study indicate that WBV may represent a feasible strategy to mitigate LST loss during the early postoperative period.

The small or null effect in other registered variables could be attributed to several factors. First, the duration of the intervention, the intensity of resistance training, and dietary protein intake could have influenced the outcomes. In patients who have undergone MBS, protein intake is often lower than recommended due to the surgery-derived reduction in gastric capacity, altered digestion and absorption, and early satiety, which may impair LST preservation. Therefore, protein supplementation could represent a useful strategy to promote muscle maintenance [19].

Regarding RMR, a differential effect was observed in the short-term (T2-T3): the WBV group did not show a significant change in absolute RMR, whereas the CG did. Although this interaction was not statistically significant, the difference in average values suggest that exercise may help blunt the decline in RMR. Similarly, over the entire study period (T1-T3), RMR decreased significantly in both groups, with no significant differences between groups (WBV: −430.6 vs. CG: −536.3 kcal/day, *p* = 0.085).

Our finding contributes to a significant gap in the literature; there is a notable lack of evidence investigating exercise and RMR in the early postoperative phase [46]. During this period, the most rapid weight loss and metabolic adaptations occur. The majority of existing studies have explored this relationship in patients awaiting surgery or in the long-term [17, 18, 47–49]. Studies found a preservation of RMR despite significant weight loss. Among the few relevant studies, the work of Jo et al. [17] offers a compelling parallel. They reported that despite substantial weight loss in both groups, only the high-intensity resistance training group successfully preserved both LST and absolute RMR. In contrast, the CG experienced a significant reduction in RMR (−328.6 ± 72.7 kcal/day) accompanied by a greater loss of LST. This clear dissociation demonstrates that exercise can decouple the expected decline in RMR from the fact of weight loss itself. The findings strengthen the physiological plausibility of our observation, indicating that early postoperative exercise may be a strategy to modulate the drastic metabolic slowdown.

### Association Between RMR and Body Composition

Our study provides comprehensive insights into the complex relationships between RMR and body composition parameters and reaffirms that LST is an important cross-sectional determinant of RMR, both before and after SG. From a physiological perspective, RMR is primarily determined by FFM, which accounts for approximately 65–90% of its variability, while FM contributes to a much lesser extent [7]. Crucially, our analysis provides a more refined view by including ALST, a direct marker of limb muscle mass and the ALST index, a key criterion for sarcopenia. By focusing on the appendicular compartment, we aimed to minimize the confounding metabolic influence of high-turnover visceral organs, thereby offering a clearer assessment of the specific relationship between skeletal muscle mass and energy expenditure. This perspective aligns with the concept that a metabolically favourable body composition is characterized not only by reduced adiposity but, critically, by the preservation of lean tissue, which plays a central role in maintaining RMR.

At baseline, strong associations were observed between RMR and LST, ALST and ALST index all adjusted for gender. Cross-sectional analysis at T2 and T3 reinforced these findings, with high R^2^ values (0.66–0.81) for all LST markers. Furthermore, we found that baseline LST parameters were significant predictors of future RMR. LST, ALST, and the ALST index at T1 all strongly predicted RMR at T2 and T3. Similar results were found in a two-year follow-up after SG [38], this indicates the need for prehabilitation programmes to be implemented before surgery.

However, a more complex picture emerged from our longitudinal analysis. The changes in RMR were not significantly predicted by the changes in LST or ALST over most time intervals, with only a trend appearing from baseline to the 6-month follow-up. This dissociation between changes in LST and changes in RMR is consistent with the broader scientific literature. Previous studies in MBS populations have similarly reported strong cross-sectional FFM-RMR relationships but weak longitudinal ones [38, 40].

This pattern suggests that while LST establishes the foundational metabolic rate, its changes may become more metabolically relevant in the longer term, while in an earlier phase other adaptive mechanisms may dominate the RMR decline following weight loss, for instance, in our longitudinal analysis changes in trunk FM demonstrated significant associations in this early phase (T1-T2).

This pattern is supported by Martin et al. [50], who demonstrated in the CALERIE study that adipose tissue loss, rather than skeletal muscle mass, was the primary driver of RMR reduction following weight loss. Galtier et al. [16] found a relationship between reduction in RMR and visceral fat area after a MBS. Another study showed a positive correlation between upper body fat [15] aligned with our significant association between trunk FM and RMR at baseline. These findings confirm the concept that not all adipose tissues are metabolically inert and trunk FM, which includes visceral and abdominal subcutaneous depots, may possess greater metabolic activity and contribute more significantly to energy expenditure than peripheral fat. This aligns with the idea that approximately 40% of the RMR reduction represents a metabolic adaptation unexplained by tissue loss alone [50, 51]. Factors such as decreased sympathetic nervous system activity, reduced thyroid hormone function, and potentially diminished brown adipose tissue might contribute to this effect [52–54]. Together, these changes help explain why maintaining weight loss remains an ongoing biological challenge. Structured exercise may help counteract these hormonal shifts by preventing marked declines in leptin, highlighting its potential role in reducing metabolic adaptation despite tissue loss.

Although some longitudinal studies suggest that RMR may partially recover or stabilize after the initial weight loss phase [9, 55], these findings remain heterogeneous and appear to depend on changes in FFM composition and organ-specific metabolic activity. This suggests that early reductions in RMR may partly reflect transient adaptive mechanisms that differ across post-operative phases.

Taken together, these observations underscore the need for further research to better characterize the evolution of RMR and body composition beyond the early postoperative period, as well as to clarify the role of exercise in modulating these adaptations. Ongoing follow-up studies in this cohort may help to further elucidate these longer-term responses.

### Clinical Implications and Feasibility of WBV Training

The positive findings observed for ALST suggest that WBV may represent a feasible exercise modality during the early postoperative period, when patients frequently present marked deconditioning, reduced exercise tolerance, and musculoskeletal limitations associated with severe obesity. Although current evidence does not indicate that WBV is superior to conventional resistance or aerobic exercise, previous studies suggest that it may provide comparable benefits for selected body composition and physical fitness outcomes while requiring lower cardiovascular and perceived effort demands [21, 56, 57]. However, the present intervention was delivered through one-to-one supervised sessions three times per week for 16 weeks, which requires substantial personnel and healthcare resources and may limit scalability in routine clinical practice. Nevertheless, from a practical perspective, individual supervision may be particularly valuable during the early postoperative period, when patients often require close guidance not only to learn exercise techniques and develop postural control, but also to build confidence, overcome movement-related fears, and adapt to the profound physical and psychological changes associated with MBS. Future studies should investigate whether a transition from initial one-to-one supervision to group-based, hybrid, or home-based WBV programmes could maintain clinical benefits while improving feasibility and cost-effectiveness. Furthermore, additional research is needed to compare different exercise modalities, WBV protocols, training doses, and progression strategies in order to identify the most effective and tolerable approaches for this population, particularly those that maximize adherence and clinical outcomes while minimizing adverse symptoms such as dizziness or exercise intolerance during the early postoperative period.

### Limitations

First, as a pilot randomized controlled trial, the sample size was not based on a formal a priori power calculation for RMR and may therefore have limited the ability to detect small-to-moderate between-group differences. Consequently, the findings should be interpreted with caution and confirmed in larger adequately powered trials.

Second, multiple body composition and metabolic outcomes were examined and no formal correction for multiple comparisons was applied. Therefore, the findings should be interpreted as exploratory and hypothesis-generating.

Third, although dietary intake and habitual physical activity were assessed and are presented in the Supplementary Material, these variables were not included in the main statistical models. Therefore, residual confounding related to lifestyle behaviours cannot be completely excluded. Importantly, no participants were treated with GLP-1 receptor agonists during the study period.

Fourth, although all assessments were performed under standardized conditions, including overnight fasting, morning testing, and verbal confirmation of compliance with pre-assessment requirements, hydration status and bladder emptying were not objectively standardized before DXA measurements. As both factors may influence DXA-derived estimates of LST, their effect on body composition measurements cannot be completely excluded.

Finally, the WBV protocol, while feasible and well tolerated in the early postoperative setting, may not provide the same anabolic stimulus as conventional resistance training.

## Conclusions

Our findings demonstrate that SG induces rapid and substantial losses in both FM and LST, accompanied by a significant reduction in RMR. In this pilot randomized controlled trial, a 16-week WBV exercise intervention attenuated losses in ALST and ALST index during the early postoperative period. Although no significant between-group differences were observed for RMR, the role of exercise in modulating postoperative metabolic adaptations warrants further investigation in larger adequately powered trials.

Regression analysis revealed that LST parameters are strong cross-sectional determinants and predictors of RMR both before and after surgery. The finding that baseline LST and ALST predict postoperative RMR provides a compelling rationale for prehabilitation programs aimed at optimizing LST prior to MBS.

The dissociation between changes in LST and changes in RMR during the early postoperative period can be attributed to shifts in fat distribution and a complex metabolic adaptation. The interplay between tissue loss and active metabolic adjustments helps explain why sustaining weight loss continues to be a persistent biological challenge. Future research should focus on longer-term, intensified resistance exercise protocols combined with nutritional strategies.

## Supplementary Information

Below is the link to the electronic supplementary material.

Supplementary Material 1 (DOCX 19.3 MB)

## Data Availability

The datasets generated during the current study are available from the corresponding author upon reasonable request.
