# Supplementary material for: Effects of Sleeve Gastrectomy and Subsequent Whole-Body Vibration Training on Resting Metabolic Rate and Body Composition: A Pilot Randomized Controlled Trial
Source: Obes Surg. 2026 Aug 19;36(11):5652–69. doi: 10.1007/s11695-026-08890-0 (PMC13714150; doi:10.1007/s11695-026-08890-0)
Supplement: Supplementary file 1 [file 11695_2026_8890_MOESM1_ESM.docx]

**SUPPLEMENTARY FILE**

**Effects of sleeve gastrectomy and subsequent whole-body vibration training on resting metabolic rate and body composition: A pilot randomized controlled trial**

**DIETARY INTAKE AND PHYSICAL ACTIVITY LEVELS**

**Table S1**. Dietary intake from baseline (T1) to 6 months post-surgery (T3). ITT analysis.

| **Parameter (Mean ±SD)** | **WBV Group** | | **Control group** | |
| --- | --- | --- | --- | --- |
|  | **T1** | **T3** | **T1** | **T3** |
| **Dietary intake** | | | | |
| **Total energy (kcal/day)** | 2578.3±862.1 | 1509.5±552.1* | 3031.8±1350.6 | 1701.5±508.7* |
| **Protein (%)** | 17.8±3.6 | 19.0±4.8 | 17.6±2.8 | 18.1±3.8 |
| **Protein (g/day)** | 117.5±64.1 | 67.1±17.1* | 126.4±41.7 | 73.6±13.2* |
| **Total fat (%)** | 40.9±7.8 | 46.7±7.9 | 42.6±5.0 | 44.1±4.7 |
| **Lipds (g/day)** | 118.4±58.1 | 80.1±37.4* | 143.4±65.4 | 84.5±29.8* |
| **Carbohydrates (%)** | 38.5±9.8 | 33.9±8.4 | 38.0±6.5 | 36.2±5.3 |
| **Carbohydrates (g/day)** | 242.3 ± 82.6 | 127.6 ± 59.2* | 295.2 ± 139.7 | 152.6 ± 51.6* |
| **Physical activity levels** | | | | |
| **PA (Kcal/week)** | 3438±1292 | 6438±2127 | 3873±384 | 4810±2279 |
| **g/**day, grams per day; kcal/day, kilocalories per day; kcal/week, kilocalories per week; IPAQ, International Physical Activity Questionnaire; PA, physical activity; T1, one week before surgery; T3, six months after surgery; WBV, whole-body vibration. p < 0.05 vs. T1 within the same group. No significant between-group differences were observed for dietary intake or physical activity variables. | | | | |

**PER PROTOCOL ANALYSIS**

**Table S2.** Effects of sleeve gastrectomy and exercise intervention (T2-T3). PP analysis.

| **Parameter (Mean ±SD)** | **WBV Group**  **n=10** | | | | **Control Group**  **n=10** | | | | **GxT** | **η²_p_** |
| --- | --- | --- | --- | --- | --- | --- | --- | --- | --- | --- |
|  | **T2** | **T3** | **Δ (T2-T3)** | **η²_p_** | **T2** | **T3** | **Δ (T2-T3)** | **η²_p_** |  |  |
| **Body composition and anthropometric variables** | | | | | | | | | | |
| **Body weight (kg)** | 122.34±16.06 | 101.28±11.11 | -21.06±7.38** | 0.901 | 110.64±10.39 | 91.13±10.06 | -19.51±3.09** | 0.978 | 0.548 | 0.020 |
| **Waist Circumference (cm)** | 112.60±10.17 | 97.00±8.25 | -15.60±8.41** | 0.793 | 112.30±9.49 | 97.00±6.09 | -15.30±6.65** | 0.855 | 0.930 | 0.000 |
| **Lean soft tissue (kg)** | 64.10±9.69 | 60.10±9.87 | -3.98±1.89** | 0.831 | 59.00±9.86 | 55.05±10.2 | -4.07±2.63** | 0.727 | 0.933 | 0.000 |
| **Lean soft tissue (%)** | 52.57±6.01 | 59.28±6.74 | 6.72±3.17** | 0.833 | 53.04±4.39 | 59.94±5.59 | 6.90±2.03** | 0.927 | 0.882 | 0.001 |
| **ALST index (kg/m²)** | 9.53±1.19 | 9.08±1.14 | -0.45±0.34* | 0.664 | 9.10±1.07 | 8.35±1.17 | -0.75±0.37** | 0.825 | 0.070 | 0.171 |
| **Fat mass (kg)** | 56.78±11.79 | 40.43±8.65 | -16.35±6.27** | 0.883 | 50.00±3.02 | 35.31±4.16 | -14.69±1.69** | 0.988 | 0.428 | 0.035 |
| **Fat mass (%)** | 46.2±6.13 | 40.0±6.69 | -6.36±2.54** | 0.875 | 45.6±5.30 | 39.1±5.82 | -6.47±1.43** | 0.958 | 0.908 | 0.001 |
| **Trunk fat (kg)** | 25.29±3.44 | 17.0±2.83 | -8.29±3.16** | 0.884 | 24.11±2.45 | 16.3±2.76 | -7.77±1.74** | 0.957 | 0.650 | 0.012 |
| **Android/Gynoid fat index** | 0.56±0.13 | 0.50±0.12 | -0.06±0.04* | 0.708 | 0.61±0.13 | 0.54±0.09 | -0.07±0.06* | 0.616 | 0.394 | 0.041 |
| **Resting metabolic rate variables** | | | | | | | | | | |
| **RMR (kcal/day)** | 1906.80±236.12 | 1831.70±245.57 | -75.10±151.39 | 0.215 | 1931.80±332.75 | 1749.10±266.68 | -182.70±104.71** | 0.774 | 0.080 | 0.160 |
| **RMR/ BW** | 15.67±1.57 | 18.14±2.11 | 2.47±1.52 | 0.745 | 17.37±1.61 | 19.17±1.67 | 1.80±1.17* | 0.726 | 0.284 | 0.064 |
| **RMR/ Fat mass** | 34.81±8.24 | 47.47±13.92 | 12.67±7.66** | 0.866 | 38.92±8.10 | 50.67±13.18 | 11.75±6.39** | 0.908 | 0.394 | 0.041 |
| **RMR/ LST** | 29.94±2.66 | 30.69±2.55 | 0.76±2.30 | 0.107 | 32.76±1.83 | 32.07±2.25 | -0.69±2.26 | 0.095 | 0.173 | 0.101 |
| A/G, Android/Gynoid ratio; ALST, Appendicular lean soft tissue; BW, Body weight; cm, centimetres; GxT, Group by time interaction; η²ₚ, Partial eta square; IQR, interquartile range; kcal, kilocalories; kg, kilograms; LST, Lean Soft tissue*; p*, significance level; RMR, Resting Metabolic Rate; SG, Sleeve Gastrectomy; T2, 7 weeks post-surgery; T3, 6 months post-surgery Δ, change; *Within group changes (p<0.05); **Within group changes (p<.001) | | | | | | | | | | |

**Table S3.** Changes from baseline (T1-T3). PP analysis.

| **Parameter (Mean ±SD)** | **WBV Group**  **n=10** | | **Control Group**  **n=10** | | **GxT** | **η²p** |
| --- | --- | --- | --- | --- | --- | --- |
|  | **Δ (T1-T3)** | **η²p** | **Δ (T1-T3)** | **η²p** |  |  |
| **Body composition and anthropometric variables** | | | | | | |
| **Body weight (kg)** | -38.37±9.84** | 0.944 | -36.97±6.14** | 0.976 | 0.707 | 0.008 |
| **Waist Circumference (cm)** | -25.70±8.47** | 0.911 | -29 ±7.44** | 0.944 | 0.367 | 0.045 |
| **Lean soft tissue (kg)** | -11.79±3.90** | 0.910 | -11.83±3.70** | 0.919 | 0.982 | 0.000 |
| **Lean soft tissue (%)** | 7.71±3.90** | 0.889 | 7.98±3.90** | 0.957 | 0.807 | 0.003 |
| **ALST index (kg/m²)** | -1.86±0.75** | 0.873 | -1.97 ± 0.49** | 0.948 | 0.715 | 0.008 |
| **Fat mass (kg)** | -25.73±6.36** | 0.948 | -24.95±2.30** | 0.992 | 0.443 | 0.033 |
| **Fat mass (%)** | -7.44±1.75** | 0.953 | -7.21±1.98** | 0.936 | 0.786 | 0.004 |
| **Trunk fat (kg)** | -13.36±3.38** | 0.945 | -13.84±2.77** | 0.965 | 0.731 | 0.007 |
| **Android/Gynoid fat index** | -0.08±0.05* | 0.759 | -0.10±0.08* | 0.655 | 0.635 | 0.013 |
| **Resting metabolic rate variables** | | | | | | |
| **RMR (kcal/day)** | -429.60±157.22** | 0.892 | -536.30±131.27** | 0.948 | 0.117 | 0.131 |
| **RMR/BW** | 1.88±1.02 | 0.791 | 1.40±1.21 | 0.599 | 0.351 | 0.048 |
| **RMR/ Fat mass** | 12.14±5.29** | 0.942 | 11.70±7.53** | 0.889 | 0.341 | 0.050 |
| **RMR/ LST** | -0.85±1.96 | 0.174 | -2.30±2.29 | 0.529 | 0.145 | 0.114 |
| A/G, Android/Gynoid ratio; ALST, Appendicular lean soft tissue; BW, Body weight; cm, centimetres; ; GxT, Group by time interaction; η²ₚ, Partial eta square;; kcal, kilocalories; kg, kilograms; LST, Lean Soft tissue; *p*, significance level; RMR, Resting Metabolic Rate; SG, Sleeve Gastrectomy; T1, one week before surgery; T3, six months post-surgery; Δ, change; *Within group changes (p<0.05); **Within group changes (p<.001) | | | | | | |

**ADJUSTED ANALYSIS**

**Table S4.** Effects of sleeve gastrectomy and exercise intervention (T2-T3). Sensitivity analyses using ANCOVA models adjusted for baseline value (T2), age, and sex. ITT analysis.

| **Outcome (T3)** | **F (Group)** | ***p*-value** | **η²p** |
| --- | --- | --- | --- |
| **Body composition and anthropometric variables** | | | |
| **Body weight (kg)** | 0.745 | 0.399 | 0.038 |
| **Waist Circumference (cm)** | 0.326 | 0.575 | 0.018 |
| **Lean soft tissue (kg)** | 2.362 | 0.141 | 0.111 |
| **Lean soft tissue (%)** | 0.387 | 0.541 | 0.020 |
| **ALST (kg)** | 7.868 | **0.011** | 0.293 |
| **ALST index (kg/m²)** | 6.855 | **0.008** | 0.318 |
| **Fat mass (kg)** | 0.030 | 0.864 | 0.002 |
| **Fat mass (%)** | 0.452 | 0.510 | 0.023 |
| **Trunk fat (kg)** | 0.054 | 0.818 | 0.003 |
| **Android/Gynoid fat index** | 0.180 | 0.676 | 0.009 |
| **Resting metabolic rate variables** | | | |
| **RMR (kcal/day)** | 2.484 | 0.131 | 0.116 |
| **RMR/BW** | 0.204 | 0.657 | 0.011 |
| **RMR/FM** | 1.475 | 0.240 | 0.072 |
| **RMR/LST** | 0.323 | 0.576 | 0.017 |
| A/G, Android/Gynoid ratio; ALST, Appendicular lean soft tissue; BW, Body weight; cm, centimetres; GxT, Group by time interaction; η²ₚ, Partial eta square; IQR, interquartile range; kcal, kilocalories; kg, kilograms; LST, Lean Soft tissue*; p*, significance level; RMR, Resting Metabolic Rate; SG, Sleeve Gastrectomy; T2, 7 weeks post-surgery; T3, 6 months post-surgery | | | |

**Table S5.** Changes from baseline (T1-T3). ANCOVA Sensitivity analyses adjusted for baseline value (T1), age and sex. ITT analysis.

| **Outcome (T3)** | **F (Group)** | ***p*-value** | **η²p** |
| --- | --- | --- | --- |
| **Body composition and anthropometric variables** | | | |
| **Body weight (kg)** | 1.334 | 0.262 | 0.000 |
| **Waist Circumference (cm)** | 0.213 | 0.650 | 0.012 |
| **Lean soft tissue (kg)** | 2.106 | 0.163 | 0.100 |
| **Lean soft tissue (%)** | 0.095 | 0.761 | 0.005 |
| **ALST (kg)** | 4.107 | 0.057 | 0.178 |
| **ALST index (kg/m²)** | 1.928 | 0.181 | 0.092 |
| **Fat mass (kg)** | 0.278 | 0.604 | 0.014 |
| **Fat mass (%)** | 0.921 | 0.349 | 0.046 |
| **Trunk fat (kg)** | 0.536 | 0.473 | 0.027 |
| **Android/Gynoid fat index** | 0.819 | 0.377 | 0.041 |
| **Resting metabolic rate variables** | | | |
| **RMR (kcal/day)** | 4.866 | **0.040** | 0.204 |
| **RMR/BW** | 0.098 | 0.757 | 0.005 |
| **RMR/FM** | 1.327 | 0.264 | 0.065 |
| **RMR/LST** | 0.232 | 0.636 | 0.012 |
| A/G, Android/Gynoid ratio; ALST, Appendicular lean soft tissue; BW, Body weight; cm, centimetres; ; GxT, Group by time interaction; η²ₚ, Partial eta square;; kcal, kilocalories; kg, kilograms; LST, Lean Soft tissue; *p*, significance level; RMR, Resting Metabolic Rate; SG, Sleeve Gastrectomy; T1, one week before surgery; T3, six months post-surgery; | | | |

**Figure S1.** Relationship between RMR and LST variables at baseline across study time points, adjusted for gender. (A) RMR T2 vs. LST T1; (B) RMR T3 vs. LST T1; (C) RMR T2 vs. ALST T1; (D) RMR T3 vs. ALST T1; (E) RMR T2 vs. ALST index T1; (F) RMR T3 vs. ALST index T1; ALST, Appendicular lean soft tissue; ALST Index, Appendicular Lean Soft Tissue Index; LST, Lean Soft Tissue p, p-value; RMR, Resting Metabolic Rate; R², coefficient of determination. T1, one week before surgery; T2, seven weeks post-surgery; T3= six months post-surgery.

**Figure S2**. Relationship between RMR and total LST variables across study time points, adjusted for gender. (A) RMR T1 vs. Total FAT T1; (B) RMR T1 vs. Fat Trunk T1; (C) RMR T1 vs. A/G index T1; (D) RMR T2 vs. Total FAT T2; (E); RMR T2 vs. Fat Trunk T2; (F) RMR T2 vs A/G index T2. A/G index, Android to Gynoid fat ratio; RMR, Resting Metabolic Rate; R², coefficient of determination T1, one week before surgery; T2, even weeks post-surgery; T3, six months post-surgery

**Figure S3.** Relationship between RMR and changes in FAT variables across study time points, adjusted for gender. (A) ΔRMR T1–T2 vs. ΔTotal FAT T1–T2; (B) ΔRMR T1–T2 vs. ΔFat Trunk T1–T2; (C) ΔRMR T1–T2 vs. A/G index T1–T2; (D) ΔRMR T2–T3 vs. Δ Total FAT T2–T3; (E) ΔRMR T2–T3 vs. Δ Fat Trunk T2–T3; (F) ΔRMR T2–T3 vs. Δ A/G index T2–T3. (G) ΔRMR T1–T3 vs. Δ Total FAT T1–T3 (H) ΔRMR T1–T3 vs. Δ Fat Trunk T1–T3 (I) ΔRMR T1–T3 vs. Δ A/G index T1–T3; p, p-value; RMR, Resting Metabolic Rate; R², coefficient of determination. T1, one week before surgery; T2, seven weeks post-surgery; T3, six months post-surgery.
